# Intervention-induced changes in neural connectivity during motor preparation may affect cortical activity at motor execution

**DOI:** 10.1038/s41598-020-64179-x

**Published:** 2020-04-30

**Authors:** Kevin B. Wilkins, Julius P. A. Dewald, Jun Yao

**Affiliations:** 1 Department of Physical Therapy and Human Movement Sciences, Northwestern University, 645 N Michigan Ave, Suite 1100, Chicago, IL 60611 USA; 2 Northwestern University Interdepartmental Neuroscience, Northwestern University, 320 E. Superior St, Chicago, IL 60611 USA; 3 Department of Biomedical Engineering, Northwestern University, 2145 Sheridan Road, Evanston, IL 60208 USA; 4 Department of Physical Medicine and Rehabilitation, Northwestern University, 345 East Superior Street, Chicago, IL 60611 USA

**Keywords:** Motor cortex, Stroke, Stroke

## Abstract

Effective interventions have demonstrated the ability to improve motor function by reengaging ipsilesional resources, which appears to be critical and feasible for hand function recovery even in individuals with severe chronic stroke. However, previous studies focus on changes in brain activity related to motor execution. How changes in motor preparation may facilitate these changes at motor execution is still unclear. To address this question, 8 individuals with severe chronic hemiparetic stroke participated in a device-assisted intervention for seven weeks. We then quantified changes in both coupling between regions during motor preparation and changes in topographical cortical activity at motor execution for both hand opening in isolation and together with the shoulder using high-density EEG. We hypothesized that intervention-induced changes in cortico-cortico interactions during motor preparation would lead to changes in activity at motor execution specifically towards an increased reliance on the ipsilesional hemisphere. In agreement with this hypothesis, we found that, following the intervention, individuals displayed a reduction in coupling from ipsilesional M1 to contralesional M1 within gamma frequencies during motor preparation for hand opening. This was followed by a reduction in activity in the contralesional primary sensorimotor cortex during motor execution. Similarly, during lifting and opening, a shift to negative coupling within ipsilesional M1 from gamma to beta frequencies was accompanied by an increase in ipsilesional primary sensorimotor cortex activity following the intervention. Together, these results show that intervention-induced changes in coupling within or between motor regions during motor preparation may affect cortical activity at execution.

## Introduction

In individuals with stroke, improvements following an intervention are at least partially mediated by neural changes. One of the more common findings for effective interventions is a return to “normal” cortical activity patterns characterized by an increased reliance on the ipsilesional hemisphere that resembles patterns observed in healthy controls^1,2^. To this point, most investigations of intervention-induced neural changes have focused on cortical activity related to motor execution^3–5^. However, the proper motor command during execution requires appropriate motor preparation. How intervention-induced changes in motor preparation may facilitate cortical changes related to execution is still unknown.

In healthy controls, the temporal-spatial feature of motor preparation related to non-visually guided hand/finger movements is characterized by a flow of information from secondary motor areas to contralateral primary motor cortex^6–9^. In addition, previous results reported decreased power (i.e., desynchronization) in beta (13–30 Hz) and mu (8–12 Hz) during motor preparation, and an association between such desynchronization and the release of the motor command^10,11^. Meanwhile, power in higher gamma frequencies (30–80 Hz) increases (i.e., synchronization) during movement preparation. Unlike lower frequencies, this increase in gamma reflects local intracortical processing rather than descending motor commands^12,13^. Considering that these different rhythms are known to operate in distinct temporal windows at different spatial scales, cross-frequency coupling may be one mechanism to transfer information across these different spatiotemporal hierarchies^14^. In support of this possibility, different types of nonlinear coupling have been found to arise during motor tasks/regions in both healthy and disease^15,16^.

Following a stroke, spatial and frequency features during motor preparation have been shown to be altered. For example, previous studies have reported overactivation of the supplementary motor area (SMA)^17^ as well as increased overlap of limb representations on the cortex^18^, which is especially pronounced in individuals with severe impairments. Post-stroke individuals also display stroke-induced changes in these neuronal oscillations during movement such as reduced beta desynchronization and changes in gamma-beta coupling between sensorimotor regions^19,20^.

Following interventions, changes occur not only at motor performance level but also in motor related brain activity. The majority of intervention studies to date have focused on cortical changes related to motor execution. We argue that understanding how changes in motor preparation may facilitate changes in motor execution is also critical. One piece of the evidence in favor of this is from Norman and colleagues, who showed that specifically training preparation-related cortical oscillations led to subsequent improvements in motor function, further cementing the crucial role for motor preparation in proper movement^21^. Given the critical role of motor preparation in performance, investigating how these frequency characteristics during motor preparation change following an intervention would provide additional details into the nature of any observed cortical reorganization.

An important caveat regarding motor preparation and execution is that it may differ based on the type of movement performed. For instance, even healthy controls show differences in motor preparation as additional joints need to be controlled for a movement^9^. This is particularly relevant in stroke since hand opening ability and reaching performance are exacerbated when combined with having to simultaneously lift at the shoulder, and these behavioral deficits are accompanied by abnormal changes in cortical activity during both motor preparation and execution. Whether an intervention can positively change cortical activity in motor preparation and execution across these different types of movements is still unclear.

This study was designed to examine how cortical changes in motor preparation may accompany changes in motor execution following an intervention that targeted hand/arm function recovery in individuals with severe chronic hemiparetic stroke. We hypothesized that intervention-induced changes in cortico-cortico interactions during motor preparation would complement changes in activity at motor execution specifically towards an increased reliance on the ipsilesional hemisphere. To test this hypothesis, we examined dynamic cortical coupling between motor regions during motor preparation and cortical activity within motor regions at motor execution for hand opening in isolation and combined with lifting at the shoulder following an effective device-assisted intervention. Through this combined approach, we explored both the interactions between regions during motor preparation and any intervention-induced activity changes. Specifically, we investigated regions that synchronize power at the same frequency (i.e., linear coupling) or across different frequencies (i.e., nonlinear coupling) because the well-established physiological underpinnings of specific frequency bands within the motor system would provide insight into the underlying neural mechanisms that may be shaping cortical activity at motor execution^22–25^.

## Methods

The EEG-data during the motor execution phase related to pure hand opening have been reported before^5^. Here, we analyzed these data during motor preparation phase in addition to the motor execution phase, to investigate the cortico-cortico interactions during motor preparation. Other data related to hand opening while lifting the arm are original.

### Stroke participants

Eight individuals with chronic hemiparetic stroke (mean age: 63.5 + 4 yrs) participated in this study. All individuals had subcortical lesions and were classified as severely impaired based on the UEFMA (UEFMA range: 11–24). Participant information and the methods of the intervention have been reported before but are listed again here^5,26^. A licensed physical therapist screened all individuals for the study based on the following inclusion criteria: severe impairment based on an UEFMA between 10 and 30 out of a total of 66, ability to undergo an MRI, no treatment involving botulinum toxin in at least 6 months, detectable EMG activity from extensor carpi radialis or extensor communis digitorum, the ability to lift the paretic arm, and the ability to give informed consent. See Table 1 for full demographics. This study was approved by the Northwestern University institutional review board. All procedures performed in studies involving human participants were in accordance with the ethical standards of the institutional and/or national research committee with the 1964 Helsinki declaration and its later amendments or comparable ethical standards. All participants gave informed written consent and consent for publication of identifying information/images in an online open-access publication.

**Table 1:** Participant demographics and clinical characteristics.

Participant	Age	Time Since Stroke (yrs)	Affected Hand	Dominant Hand	UE FMA	CMSA
P01	64	9	R	R	23	3
P02	62	8	L	R	12	3
P03	68	3	L	R	17	3
P04	61	22	L	R	11	3
P05	61	13	L	R	24	3
P06	70	20	R	R	13	3
P07	59	6	R	R	24	3
P08	63	9	R	R	22	4

Note: Upper Extremity Fugl Meyer Assessment (UE FMA); Chedoke McMaster Stroke Assessment (CMSA).

### Intervention design

**Figure 1:**
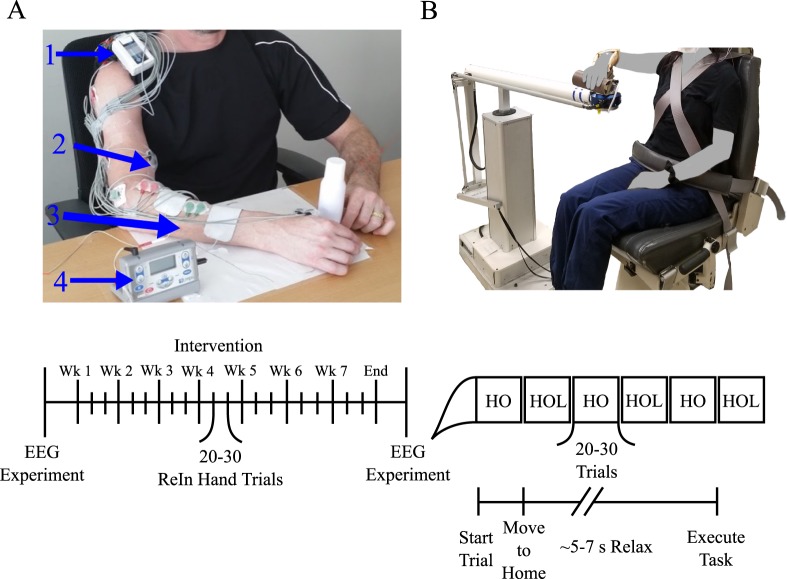
Depiction of the setup for the intervention and EEG experiment. (**A**) ReIn-Hand Device setup, including (1) EMG recording unit, (2) surface EMG electrode, (3) stimulation electrode, and (4) the FES device. (**B**) Setup on the ACT-3D robot for the EEG experiment. Timeline for the intervention (Left) and EEG experiment (Right) are depicted on the bottom. HO = Hand Opening on table; HOL = Hand Opening while Lifting.

Individuals took part in a 7-week intervention with 3 sessions per week lasting approximately 2 hours as described previously^5,27^. During each session, individuals completed 20–30 trials of a reach-grasp-retrieve-release task with a jar while seated at a table. In order to execute this task for these individuals with severe motor impairments, a novel EMG-FES device was developed (see Fig. 1A)^28^. The novelty of this device is that it uses EMG features to detect the user’s intent to open the hand even during lifting and reaching movements that would typically increase the presence of the flexion synergy at the elbow^29^, wrist, and fingers and restrict hand opening ability^30,31^. Once it detects an opening-intent, the device triggers an Empi transcutaneous electrical neuro-stimulation device (Vista, CA, USA) with stimulation electrodes on the wrist and finger extensors^5,27^ to open the paretic hand. The ReIn-Hand device allows for intuitive control since the same muscles the participant is attempting to drive (i.e. finger/wrist extensors) are being used to control the stimulation^28^.

### EEG experiment and behavioral task

Before starting and after completion of the 7-week intervention, individuals participated in an EEG experiment following a similar paradigm as described previously^5,9,27^ (see Fig. 1B). In this paradigm, a robot, the ACT3D, was used to control the shoulder abduction loads that were applied to each participant. The ACT^3D^ is an admittance controlled robotic device instrumented with a 6 degree of freedom load cell (JR^3^ Inc., Woodland, CA) that is attached to a Biodex chair (Biodex Medical Systems, Shirley, NY), which allowed the manipulation of the perceived weight of the participant’s arm. The participant’s paretic arm was strapped to an orthosis supporting the hand and forearm, which was attached to the end effector of the ACT^3D^. At the beginning of each trial, participants moved their hand to a home position which required the following configuration: 85° shoulder abduction, 40° shoulder flexion, and 90° elbow flexion. Once the participant reached this position, they relaxed for at least several seconds to establish a baseline recording and then proceeded to perform one of 2 movements: (1) maximum hand opening with the arm resting on a haptic table created by the ACT^3D^ robot, or (2) maximum hand opening while lifting against 50% of their maximum shoulder abduction (SABD) force, as stated in our previous publication^5,27^. This shoulder abduction level was used since it is roughly equivalent to the weight of the limb in moderate to severe chronic stroke participants, thus making it a functionally relevant shoulder abduction level for translation to many activities of daily living that require simultaneously using the hand while lifting the arm. The experiment consisted of 60 to 70 trials for each condition/movement. These trials were broken into blocks of 20 to 30 trials that were randomized.

Participants were instrumented with a 160-channel EEG system with active electrodes (Biosemi, Inc., Active II, Amsterdam, The Netherlands). Prior to beginning the experiment, impedances were checked to ensure they were below 50 kΩ. EMGs were simultaneously recorded over the wrist/finger extensor, wrist/finger flexor, and deltoid of the paretic arm with the Biosemi system.

The sampling frequency of both the EEG and EMG data was 2048 Hz. Once the EEG electrodes were placed on the participant’s head, the positions of each electrode were recorded relative to the nasion and pre-auricular notches with a Polaris Krios scanner (NDI, Ontario, Canada).

### Structural imaging of the brain

Participants participated in MRI scans at Northwestern University’s Center for Translation Imaging on a 3 Tesla Siemens Prisma scanner using a 64-channel head coil. The parameters used have been described before^5,9^: An MP-RAGE sequence MP-RAGE sequence (TR = 2.3 s, TE = 2.94 ms, FOV = 256 × 256 mm^2^) was used to acquire T1-scans with a voxel resolution of 1 mm^3^.

### Data analysis for cortico-cortical connectivity during motor preparation

To investigate the cortico-cortico connectivity, we used dynamic causal modeling for induced responses (DCM-IR)^32^ to model the task-related time-varying changes in power both within and across a range of frequencies by estimating the coupling parameters within and between sources in a network. This approach has been used in previous hand movement tasks to elucidate the dynamic interactions within a motor network^6,9,33,34^. The methods below have been previously described^9,27^.

#### Definition of model space

Our motor network model consisted of 5 ROIs, including bilateral primary motor cortex (M1), bilateral premotor cortex (PM), and supplementary motor area (SMA). We chose these 5 ROIs for cortico-cortical-connectivity analysis for motor preparation because all of them showed confirmed roles in motor preparation^35^. Although other regions like dorsolateral prefrontal cortex and posterior parietal cortex have been shown to possibly be involved in motor preparation, current evidence is inclusive. Therefore, they are not included in our ROI for DCM-IR model. Locations of each of these regions were adapted from the Human Motor Area Template (HMAT)^36^ and are shown in Table 2. Bilateral primary somatosensory cortices were not included to reduce the computational demand and complexity of the model. Bilateral SMAs were treated as a single source due to their mesial position on the cortices. SMA also served as the input to the modelled network. It was chosen due to its critical role in motor preparation during self-initiated motor tasks, and has previously been demonstrated to be an appropriate input for self-initiated motor tasks using DCM-IR^6,9,33,34^.

**Table 2:** Coordinates of motor network.

Sources	MNI-Coordinates (X,Y,Z)
Left M1	−37 −26 60
Right M1	37 −26 60
Left PM	−35 −4 60
Right PM	35 −4 60
SMA	−2 −7 60

Note: Coordinates were adapted from Mayka *et al*., 2006.

Different within- and cross-frequency connections between these 5 sources were used to create 12 models as shown in Supplementary Figure 1 which have successfully been used before in a similar motor task in healthy controls^33^. These 12 models were separated into 2 groups. Group 1 (models 1 to 6) allowed nonlinear and linear extrinsic (between region), but only linear intrinsic (within region) connections. Group 2 (models 7 to 12) allowed both nonlinear and linear connections for both extrinsic and intrinsic connections. Within each group, the 6 models consisted of 1 fully connected model, and the other 5 models missing 1 or 2 connections that were from one premotor area (PM) to either the other PM or to M1. Using this model, we tested the importance of nonlinear frequency interactions within regions as well as the importance of various connections to premotor regions.

#### DCM preprocessing

EEG data were preprocessed in SPM12. EEG time-series data were bandpass filtered between 1 and 50 Hz, segmented into trials (−2200 to +500 ms with 0 ms indicating EMG onset), and baseline corrected. Trials exhibiting artifacts were removed. Trials free from artifact (47.7 trials  per condition on average) were then projected to 5 chosen ROIs (see below) using an equivalent current dipole model^32^. A Morlet wavelet transform with a wavelet number of 7 was used to calculate the spectrogram between 4 and 48 Hz. The spectrogram was then averaged over all trials, cropped between −1000 to 0 ms, and then baseline-corrected by subtracting the power from −1000 to -833 ms at each frequency bin. The input from SMA to the whole network was modelled as a gamma function with a peak at 400 ms prior to EMG onset with a dispersion of 400 ms. These values were chosen in order to capture the peak of the bereitschaftspotential during a self-initiated movement^37^. The model simulation was restricted to the time leading up to EMG onset (−1000 to 0 ms) to capture purely the motor preparation and command, rather than any potential sensory feedback related to the task. The dimensionality of the spectrogram was then reduced to four modes using singular value decomposition (SVD). The four modes preserved >96% of the data variance on average. This dimensionality reduction both reduced the computational demand of the model inversion and denoised the data.

#### Calculation of coupling parameters

For each of the models shown in Supplementary Fig. 1, the dynamics of the spectrogram were evaluated using the following equation for each model described above:

$$\tau \dot{g}(t)=\tau \,[\begin{array}{c}{\dot{g}}_{1}\\ \vdots \\ {\dot{g}}_{J}\end{array}]=[\begin{array}{ccc}{A}_{11} & \ldots  & {A}_{1J}\\ \vdots  & \ddots  & \vdots \\ {A}_{J1} & \ldots  & {A}_{JJ}\end{array}]g(t)+[\begin{array}{c}{C}_{1}\\ \vdots \\ {C}_{J}\end{array}]u(t),$$

where the vector *g* represents the instantaneous power at a specific timepoint and $\dot{g}$ represents its first derivative at each of the modes (results of SVD) for each of the sources in the motor network. The A matrix contains the coupling parameters within and across different modes between any 2 regions within the *J* = 5 regions, and the C matrix contains the weights of the extrinsic input $u$ from SMA. Each of the elements in the coupling A matrix refers to the influence of power at a specific frequency in one ROI on the power at another frequency in another ROI. Positive coupling suggests that changes in power in the first frequency and region lead to the same directional power change in the second frequency and region. Meanwhile, negative coupling suggests that changes in power in the first frequency and region lead to the opposite directional power change in the second frequency and region. τ is a scaling factor and *t* represents time. Using the above equation and the output of the SVD, the DCM-IR method optimizes the A and C matrices to best describe the spectrogram of the measured data. The quality of a model and the estimated A and C matrices was quantified by the accounted variance from the predicted spectrogram.

#### Bayesian model selection

Bayesian model selection (BMS) with random effects^38^ was used to compare the 12 models described above for both the hand opening and the simultaneous lifting and opening conditions using the data from all of the participants. BMS with random effects was chosen since it is better equipped to handle potential heterogeneity associated with the study of a diseased population such as stroke^38^, and it contains a complexity term that penalizes a model based on the number of parameters it uses. The winning model, which was then used for further analysis of intervention-induced changes, was chosen based on the highest posterior exceedance probability.

#### Inference on coupling parameters

Predicted spectra and A matrices from the four modes were projected back to frequency domain allowing for characterization of the coupling parameters as a function of frequency for the winning model. The coupling matrices for each participant were further smoothed with a Gaussian kernel (full-width half-maximum of 8 Hz) for each condition. These matrices include the coupling values for each connection and frequency pair (both within- and cross-frequency).

### Data analysis for cortical topography during motor execution

EEG data analysis for cortical topography have been previously described^5,27^. EEG data were low pass filtered at 70 Hz. The filtered data was segmented from −2200 to +200 ms relative to EMG onset with a baseline correction from −2180 to −2050 ms using Brain Vision Analyzer 2 software (Brain Products, Gilching, Germany). The same data exhibiting artifacts from the previous analysis were discarded and the clean trials were ensemble averaged and down-sampled to 256 Hz. A boundary element method model was then computed in Curry 6 (Compumedics Neuroscan Ltd., El Paso, Tx) for each participant based on their MRI and the recorded electrode positions. Using this subject-specific head model, the cortical current density strength (μA/mm^2^) was calculated using the standardized low-resolution electromagnetic brain tomography (sLORETA) inverse method over the time period −150 to −100 ms prior to EMG onset. Analysis on cortical activity was limited to regions of interest within the sensorimotor cortex. The ROIs analyzed included bilateral primary sensorimotor cortices (primary motor cortex (M1) combined with primary somatosensory cortex (S1)) and secondary motor cortices (supplementary motor area (SMA) combined with premotor cortex (PM)). These regions were chosen as ROIs for motor execution because they all have confirmed descending pathways to upper limb muscles.

We quantified a cortical activation ratio $CAR=\,\frac{{\sum }_{1}^{N}{S}_{n}}{{\sum }_{1}^{M}{S}_{m}}$ for each of the 4 ROIs, as discussed previously^5^. N are the indices of all the nodes in a specific ROI (each node is separated by 3 mm), and M are the indices of all the nodes in the entire sensorimotor cortex. S_n_ is the current density strength as calculated by sLORETA at a particular node. Overall, CAR is a measure of the relative strength of activity observed in one ROI normalized by the total strength of activity across the whole sensorimotor cortex.

### Statistical analysis for cortical activity

Statistics for the analyzed data were calculated using SPM and SPSS (IBM, V24). For the coupling parameters from the DCM analysis, T-statistics were used to calculate statistical parametric maps separately for each connection and condition pre/post intervention. Significance for intervention-induced changes in specific coupling parameters was set at *p* < 0.05 with family wise error (FWE) correction. For the cortical activity ratio during motor execution, a 2 (time) × 2 (task) × 4 (region) repeated measures ANOVA was performed after checking the data did not violate Mauchly’s sphericity test. Significant interactions in the ANOVA were followed up with post-hoc paired t-tests. Significance was set a *p* < 0.05.

### Ethical approval

All procedures performed in studies involving human participants were in accordance with the ethical standards of the institutional and/or national research committee with the 1964 Helsinki declaration and its later amendments or comparable ethical standards. All participants provided written informed consent.

## Results

Individuals showed an improvement in the score of the Box and Block test (1.9 block increase on average; *p* = 0.03) as well as an increase in active range of motion of the fingers (9.9° increase on average; *p* = 0.03) following the intervention based on a Wilcoxon signed rank test as reported previously^5,26^. The results included here will focus on the observed cortical neural changes pre- versus post-intervention.

### Bayesian model selection and model fit

**Figure 2:**
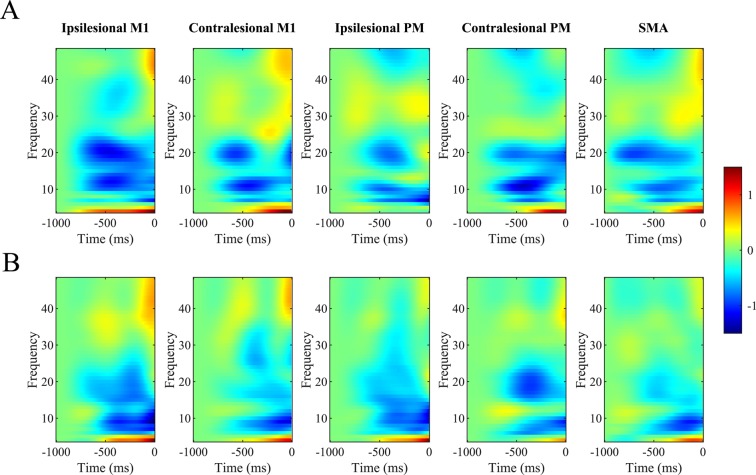
Averaged Time-Frequency plot for each source in the motor network for (**A**) Hand Opening on the table and (**B**) Hand Opening while Lifting across all participants. Red indicates increases in power relative to baseline and blue indicates decreases in power relative to baseline. 0ms represents EMG onset.

In order to determine any intervention-induced changes in connectivity, we first had to evaluate which DCM model tested best explained the observed data. BMS with random effects clearly preferred model 12 for each condition (see Supplementary Table 1), which had full connections between the 5 motor regions of interest and allowed both within- and cross-frequency interactions for intrinsic and extrinsic connections. Exceedance probabilities were 0.973 for the opening condition and 0.975 for the simultaneous lifting and opening condition. Supplementary Fig. 2 depicts both the observed and predicted spectrograms in each of the 5 motor regions using the winning model for one participant during the hand opening condition. Comparison of these two spectrograms shows the overall similarity between the observed data (i.e., power changes over time) and the model-predicted data. Overall, this model explained ~85% of the original spectral variance for each condition, indicating that it was suitable for evaluating any intervention-induced changes. Figure 2 shows the group averaged time-frequency plot as measured by the DCM of the winning model for the two tasks.

### Intervention-induced changes in coupling parameters

**Figure 3:**
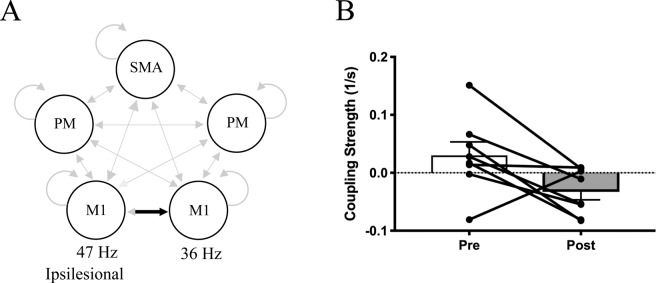
Reduced interhemispheric coupling following the intervention during motor preparation for Hand Opening on the table. (**A**) Schematic of significant decreases in coupling (black) from ipsilesional M1 (47 Hz) to contralesional M1 (36 Hz) within the motor network (light gray) following the intervention. None of the other region interactions (light gray arrows) showed significant changes pre to post intervention. (**B**) Average coupling strength data with individual data overlaid pre/post intervention for the connection depicted in A. 6 out of 8 participants demonstrate a reduction in coupling strength for this M1-M1 connection within gamma frequencies. Positive values indicate positive coupling, while negative values indicate negative coupling. Error bars represent SEM.

Once we determined the model that best explained the observed data, we examined whether any intervention-induced changes in connectivity for any of the region-region connections during motor preparation occurred. We found that the intervention induced two significant changes in the coupling parameters, one for each motor task. After the intervention, participants demonstrated significantly less positive coupling from ipsilesional M1 to contralesional M1 in gamma frequencies (47 Hz → 36 Hz) during hand opening (see Fig. 3A). When looking at the individual coupling values for this particular regional coupling, we found that prior to the intervention, 6 out of 8 participants demonstrated positive coupling values, indicating that increases in gamma in ipsilesional M1 led to increases in gamma in contralesional M1 (see Fig. 3B). However, following the intervention, 5 out of 8 participants showed zero or negative coupling, indicating that increases in gamma in ipsilesional M1 no longer led to increases in gamma in contralesional M1 (see Fig. 3B).

**Figure 4:**
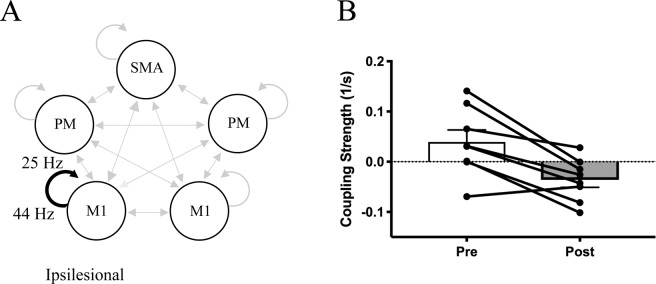
Altered intrinsic coupling within ipsilesional motor cortex following the intervention during motor preparation for Hand Opening while Lifting. (**A**) Schematic of significant decreases in intrinsic coupling (black) in ipsilesional M1 (44 Hz to 25 Hz) within the motor network (light gray) following the intervention. None of the other region interactions (light gray arrows) showed significant changes pre to post intervention. (**B**) Average coupling strength data with individual data overlaid pre/post intervention for the connection depicted in A. 7 out of 8 participants demonstrate negative coupling within ipsilesional M1 from gamma to beta frequencies following the intervention. Positive values indicate positive coupling, while negative values indicate negative coupling. Error bars represent SEM.

For the task of simultaneous lifting and opening, the intervention induced significantly more negative coupling within ipsilesional M1 from gamma to beta (44 Hz → 25 Hz) (see Fig. 4A). When looking at the individual coupling values for this particular regional coupling, we found that prior to the intervention, 6 out of 8 participants showed positive coupling values, indicating that increases in gamma power within ipsilesional M1 led to subsequent increases in beta power also within ipsilesional M1 (see Fig. 4B). However, following the intervention, 7 out of 8 participants showed negative coupling, indicating that increases in gamma power within ipsilesional M1 led to subsequent decreases in beta power within ipsilesional M1 (see Fig. 4B).

### Changes in cortical activity ratio (CAR)

**Figure 5:**
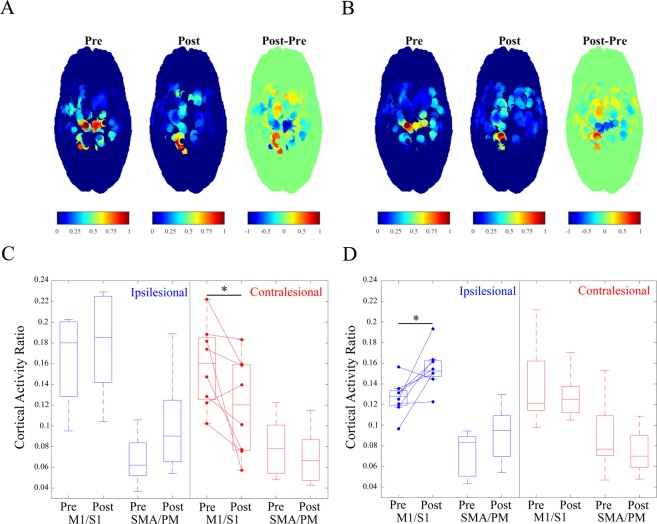
Cortical changes at movement execution following the intervention. (**A**) and (**B**) depict current source density plots across the sensorimotor cortex Pre-Intervention (Left), Post-Intervention (Middle), and the observed intervention-induced difference (Right) for (**A**) Hand Opening on the table and (**B**) Hand Opening while Lifting in one participant. The Left hemisphere is the lesioned hemisphere. (**C**) and (**D**) contain box plots depicting cortical activity ratio Pre/Post Intervention for (**C**) Hand Opening on the table and (**D**) Hand Opening while Lifting. Regions include combined primary motor cortex and primary sensory cortex (M1/S1) and combined supplementary motor area and premotor area (SMA/PM) for both the ipsilesional (Blue) and contralesional (Red) hemispheres. Participants demonstrated a decrease in contralesional primary sensorimotor cortex activity for hand opening following the intervention (left) and an increase in ipsilesional primary sensorimotor cortex activity for hand opening while lifting following the intervention (right). * indicates p < 0.05.

We then tested for any intervention-induced changes in cortical activity for the 2 tasks related to motor execution. Figure 5A,B show an example of one participant’s change in cortical activity for the two tasks following the intervention. To compare changes across the group, a 2 (time) × 2 (task) × 4 (region) repeated measures ANOVA found a significant Time * Task (*F*[1,7] = 8.03, *p* = 0.025, η_p_^2^ = 0.53) and Time * Region (*F*[3,21] = 4.64, *p* = 0.012, η_p_^2^ = 0.40) interaction. Post hoc paired t-tests found that following the intervention there was a significant decrease in the cortical activation in contralesional M1/S1 (*p* = 0.042) during hand opening (See Fig. 5C). For the simultaneous lifting and opening condition, a significant increase in cortical activation in ipsilesional M1/S1 (*p* = 0.025) was observed (See Fig. 5D).

We tested for any associated between the observed intervention-induced changes in CAR compared the changes in coupling parameters using a Spearman correlation for the two tasks. For hand opening, the association between change in contralesional M1/S1 CAR and gamma-gamma coupling from contralesional to ipsilesional M1 showed an r = 0.69, but this did not reach significance (*p* = 0.069; Supplementary Fig. 4A). Similarly, the association between change in ipsilesional M1/S1 CAR and change in gamma-beta ipsilesional M1coupling showed an r = 0.69, but this did not reach significance (*p* = 0.069; Supplementary Fig. 4B).

## Discussion

The current study quantified changes in both cortico-cortico coupling during motor preparation and cortical topography at motor execution related to paretic hand opening with and without coordination of shoulder abduction following a device-assisted intervention. Overall, we observed that intervention-induced changes in coupling during motor preparation followed similar patterns as the changes in cortical activity at motor execution across the group for the two tasks. For hand opening, this was characterized by a reduction in coupling with contralesional M1 during motor preparation and similarly decreased activity in contralesional M1 at motor execution. For hand opening while lifting, this was characterized by a change in coupling within ipsilesional motor cortex during motor preparation followed by an increase in activity in ipsilesional motor cortex at execution.

### Intervention-induced changes related to paretic hand opening

As we reported previously, participants demonstrated decreased contralesional primary sensorimotor activity related to paretic hand opening following the intervention^5^. In this study, we further demonstrated that following the intervention, these individuals demonstrated a reduction in coupling from ipsilesional M1 to contralesional M1 within gamma frequencies.

In healthy controls, gamma band power increases in contralateral primary sensorimotor cortex just before the onset and during movement^39,40^. This increase in gamma power has been shown to facilitate movement in studies using transcranial alternation current stimulation (tACS) to artificially increase gamma power over the contralateral primary sensorimotor cortex^41–43^. Typically, gamma is associated with local intracortical processing, particularly within GABAergic interneuronal circuits^12,13^, with increases in gamma power related to movement associated with decreases in GABA_A_^42^. However, gamma synchronization is usually confined to a small area in the contralateral primary sensorimotor cortex in healthy controls^39,44^, rather than bilateral increases in gamma during movement as observed here prior to the intervention.

Before the intervention, individuals with severe chronic stroke showed positive gamma coupling (as demonstrated by the positive coupling shown in Fig. 3B) from ipsilesional M1 to contralesional M1. This potentially abnormal initial positive gamma coupling between ipsilesional and contralesional M1 may indicate abnormal intercortical communication between the two hemispheres (via callosal or subcortical means^45^) given gamma’s role in integrating long-range interregional communication^13,46^ and may give rise to the initial increased contralesional activity in these individuals. This is supported by previous findings in stroke showing weaker GABAergic inhibition from the ipsilesional to the contralesional hemisphere following stroke^47,48^, and that this imbalance is associated with greater abnormal contralesional activity during paretic hand movements^49^.

Following the intervention, we found that the abnormal interhemispheric gamma coupling decreased in 6 out of 8 participants (see Fig. 3B), which supports the intervention-induced reduction in contralesional activity in the primary sensorimotor cortex (see Fig. 5C). The intervention-induced decrease in positive gamma coupling between ipsilesional and contralesional M1 may underlie the subsequent decrease in CAR in contralesional primary sensorimotor cortex and reflect a return to a more normal state as observed in healthy controls.

### Intervention-induced changes related to paretic hand opening while lifting

For the simultaneous lifting and opening task, we observed altered coupling within ipsilesional M1 in which the intervention induced a shift from positive to negative coupling between gamma and beta within ipsilesional M1 during motor preparation (see Fig. 4B). This change was accompanied by a subsequent increase in ipsilesional primary sensorimotor cortex activity at motor execution following the intervention. The observed intervention-induced change in cross-frequency coupling is particularly relevant since gamma and beta power are typically inversely related in healthy controls during movement^25^. Whereas gamma tends to increase in power prior to and during the onset of movement, beta decreases in power^40,50^. This decrease in beta power during motor preparation in healthy controls has been linked with the reduction of inhibition in M1 just prior to movement to release the motor command^10,51^. Consequently, movements executed during elevated beta synchrony are slower^52^, and increasing beta power using tACS has been shown to impair movement in healthy controls^41,53^. Importantly, stroke individuals show less decreases in beta during movement compared to controls^19^, and persistence of inhibition in ipsilesional M1 during motor preparation^54^. Therefore, the shift from positive to negative coupling between gamma and beta in ipsilesional M1 following the intervention may reflect a return to a more typical pattern seen in healthy controls.

Prior to the intervention, individuals with severe chronic stroke demonstrated increased gamma power associated with increased beta power in ipsilesional M1 (as demonstrated by positive coupling between gamma and beta power in 6/8 participants, see Fig. 4B). This positive coupling shifted to negative after the intervention, where increases in gamma power were associated with decreases in beta power. Importantly, decreases in beta power are inversely related to BOLD activity in the sensorimotor cortex, suggesting some interplay or association between these two physiological processes^55,56^. Given beta’s role in descending layer V pyramidal neurons^57,58^, the observed intervention-induced increase in CAR may reflect increased drive and use of remaining descending ipsilesional motor resources during the lifting and opening condition following the intervention, rather than purely an intracortical change. This is significant since reliance on descending tracts from the contralesional hemisphere has been linked with synergy-induced impairments^59,60^, particularly during tasks involving lifting at the shoulder^29,61,62^, as examined here. Meanwhile, increased use of descending corticospinal tract from the ipsilesional hemisphere has been shown to be crucial for improved function following stroke^63,64^, especially for independent control of multijoint movements of the upper extreminty^65^. Although contralesional corticobulbar pathways can support more proximal paretic arm movements such as reaching^66^, they do not offer sufficient control of independent joints during multijoint movements^62,67,68^. Thus, the ability to maintain ipsilesional recruitment during combined shoulder-hand tasks is critical for potential functional improvement since ipsilesional corticospinal tract, unlike contralesional corticobulbar tracts, has more specific branching in the spinal cord that allows for independent control of the different parts of the arm^68–70^.

### Implications for clinical practice

Due to the lack of control group, it is impossible to compare how this ReIn-Hand intervention compares to other common forms of therapy for stroke rehabilitation. However, a major implication of this work is that it demonstrates the feasibility for individuals with severe motor impairments in the chronic state to reengage remaining ipsilesional resources when the paretic arm is involved using an assistive device. This is crucial since although contralesional resources can help individuals compensate following a stroke, they are limited in their efficacy due to extensive branching of ipsilateral-projecting motor pathways and insufficient innervation to the paretic hand. The lack of capacity is especially pronounced during multi-joint movements involving the hand and the shoulder in which many individuals with stroke succumb to the flexion synergy due to reliance on these limited ipsilateral-projecting motor pathways. The possibility to reengage ipsilesional resources suggests that interventions should still be targeting to reestablish “normal” movement patterns through neural recovery, at least to some extent, rather than focusing purely on compensatory movement patterns. Additionally, the fact that the individuals studied here were in the chronic stage (over 11 years post-stroke on average) advocates for the continued rehabilitation of individuals even after the acute post-stroke phase.

### Limitations

One of the major limitations of the current study is the small sample size (N = 8). However, these participants are fairly homogeneous in that they are each severely impaired (FMA: 10–24; Chedoke McMaster: 3–4) with subcortical lesions impacting the posterior limb of the internal capsule and are in the chronic phase. The robotic setup during the EEG experiment also allowed for a well-controlled environment to investigate cortical activity related to the paretic arm and hand, thus minimizing potential session to session variability. Due to the smaller sample size, we decided to focus on the effects of the intervention on the two tasks separately rather than whether there was a potential interaction between the two tasks. This decision was motivated by our primary goal of investigating how an intervention would change motor preparation and execution for a particular movement.

The current study also lacked a control group to compare against the current intervention. Consequently, we cannot tease apart what may be specific to the tested intervention compared to other potential trainings. This is particularly relevant for any conclusions made from this intervention regarding whether the neural changes are indicative of neurological recovery or rather just training or exercise-related changes. Given that this is a relatively older and more sedentary cohort, the observed changes may be more reflective of involvement of some sort of training rather than specifically to the use of an EMG-driven FES combined with task-specific training. However, we argue that since participants in this study are all in the chronic stage, it is unlikely the observed changes here were spontaneous in nature. It is also unlikely that the observed results are simply due to training with the non-dominant hand since both individuals whose paretic hand matched their dominant hand and those whose paretic hand was their nondominant hand showed the observed neural changes. In fact, individuals who trained with their dominant hand showed larger changes on average for both DCM coupling parameters and CAR on both tasks. Future studies should include high-resolution quantification of kinematics though in order to disentangle true neurological recovery in which “normal” movement patterns are restored compared to new compensatory movement patterns.

There are numerous methods to analyze connectivity besides DCM-IR when measuring EEG. Methods such as correlation or coherence between two time-series are the most commonly used metrics. However, unlike DCM-IR, these are limited to within-frequency relationships. Methods evaluating both within and cross-frequency relationships are rarer, including bicoherence, n:m phase synchrony, and multi-spectral phase coherence methods. However, these methods either do not evaluate directionality, like correlations and coherence, or do not directly link to cortical regions. DCM-IR allowed us to evaluate both linear and nonlinear relationships between regions within a network, while also looking at directionality. However, DCM-IR only examines the temporal dynamics of power, but does not contain information of phase, which has been shown to play a potential role in cortical communication^71^. Additionally, the DCM-IR method uses an equivalent current dipole (ECD), which may not have the same accuracy as other inverse calculation methods such as the sLORETA that was used in the cortical activity analysis in this paper. However, these methods were chosen separately to best account for the type of data for each analysis (i.e., single trial vs. ensemble-averaged) and question at hand. The ECD was used for the DCM analysis since the goal was to capture each ROI in the model as a single dipole, in which case sLORETA would be of limited additional value. Meanwhile, in the CAR analysis we could take advantage of the nature of current density models that are not constrained to a single dipole. Lastly, DCM is also limited by the models tested. This means that we cannot make any assertions regarding potentially changes or lack of changes for regions not included in the analysis. This is especially relevant for regions such as S1 and the parietal cortex which have been previously shown to be implicated in post-stroke impairment and recovery.

We purposefully chose to analyze a different set of regions for the motor preparation DCM analysis compared to the motor execution CAR analysis based on prior knowledge regarding regions relative to motor preparation and regions that have descending tracts innervated to upper limb muscles. Although this difference causes difficulty in results interpretation, we still found agreements between results obtained using these 2 methods, further strengthening the coherence between results in preparation and execution. It is worth noting though, that since both M1/S1 have descending motor fibers and reciprocal cortico-cortico connections, it is impossible to differentiate whether the changes in CAR for M1/S1 reflect changes in efferent commands or M1-S1 interactions. This is especially relevant since we previously reported that this intervention led to sensory clinical improvements as well as gray matter changes in thalamus and sensorimotor cortices^5,26^, and FES has been shown to activate S1 based on fMRI results^72,73^. It is possible, if not likely, that the sensory changes from the FES facilitated the observed long-term motor execution-related cortical changes. However, the focus of this study is to investigate how intervention-induced changes between motor preparation and execution. How the intervention induces sensory and motor changes interactively is beyond the scope of this paper.

EEG is inherently limited in its spatial resolution, particularly in comparison to fMRI. However, using our high-density EEG setup, along with the sLORETA inverse calculation based on subject-specific boundary element models created with individual’s anatomical MRI, we have demonstrated a resolution of roughly 5 mm^74,75^. This is suitable for distinguishing primary sensorimotor cortex and secondary motor areas as investigated here in our topographical analysis and allowed us to investigate multi-joint movements which would have been impractical inside an MRI scanner. Meanwhile, the source reconstruction in DCM is based on an equivalent current dipole (ECD) method using the coordinates chosen based on our previous cortical activity findings in combination with standardized locations from the literature. Although these dipoles capture a smoothed portion of the cortex, it is possible that additional activity is not captured within the 5 regions investigated. It is worth noting that these 2 independent inverse approaches (sLORETA and ECD) yielded complementary results in the regions investigated, which we believe adds additional confidence that what we are capturing is a true neurophysiological response and not an artifact of the analysis.

Finally, we would like to point out that there is no causal evidence here that the intervention-induced changes in coupling during motor preparation lead to the changes in cortical activity at movement execution. Rather, we have found that there is a complementary relationship between the two in which similar changes observed during motor preparation accompany those seen at execution at the group level.

## Conclusion

The current study demonstrates that intervention-induced changes in coupling both within and between motor regions during motor preparation complement changes in cortical activity at motor execution and that this holds true for both the hand in isolation and in multi-joint movements of the arm. Additionally, use of DCM allowed us to disentangle the roles of different frequency interactions during motor planning that facilitate subsequent changes in cortical activity at motor execution. Together, these findings support the notion that changes in cortico-cortico interactions during motor preparation may lead to corresponding changes in focal cortical activity.

## Supplementary information

Supplementary Video 1.

Supplementary Video 2.

Supplementary Materials.

## Data Availability

Data is available upon request.
