# Supplementary material for: Intervention-induced changes in neural connectivity during motor preparation may affect cortical activity at motor execution
Source: Sci Rep. 2020 Apr 30;10:7326. doi: 10.1038/s41598-020-64179-x (PMC7193567; doi:10.1038/s41598-020-64179-x)
Supplement: Supplementary file 3 — Supplementary Materials. [file 41598_2020_64179_MOESM3_ESM.docx]

Title: Intervention-induced changes in neural connectivity during motor preparation may affect cortical activity at motor execution

Kevin B. Wilkins^a,b^, Julius P.A. Dewald^a,b,c,d^, *Jun Yao^a,b^

Supplementary Figure 1. Models tested for DCM analysis. Models 1-6 allow only linear intrinsic connections and both nonlinear and linear extrinsic connections, while Models 7-12 allow both nonlinear and linear intrinsic and extrinsic connections. Individual models differ in interhemispheric connections allowed between M1 and PM regions. Dashed lines indicate only linear connections allowed, while solid lines indicate both linear and nonlinear connections allowed. The left side is the lesioned side.

**Supplementary Table 1. Exceedance probabilities for each model**

| Models | Open | Lift + Open |
| --- | --- | --- |
| 1 | 0.0018 | 0.0022 |
| 2 | 0.002 | 0.0024 |
| 3 | 0.0028 | 0.0017 |
| 4 | 0.0023 | 0.0022 |
| 5 | 0.0021 | 0.0013 |
| 6 | 0.0022 | 0.002 |
| 7 | 0.0056 | 0.0029 |
| 8 | 0.0021 | 0.0037 |
| 9 | 0.0019 | 0.0026 |
| 10 | 0.0021 | 0.0024 |
| 11 | 0.0025 | 0.0018 |
| 12 | 0.9726 | 0.9748 |


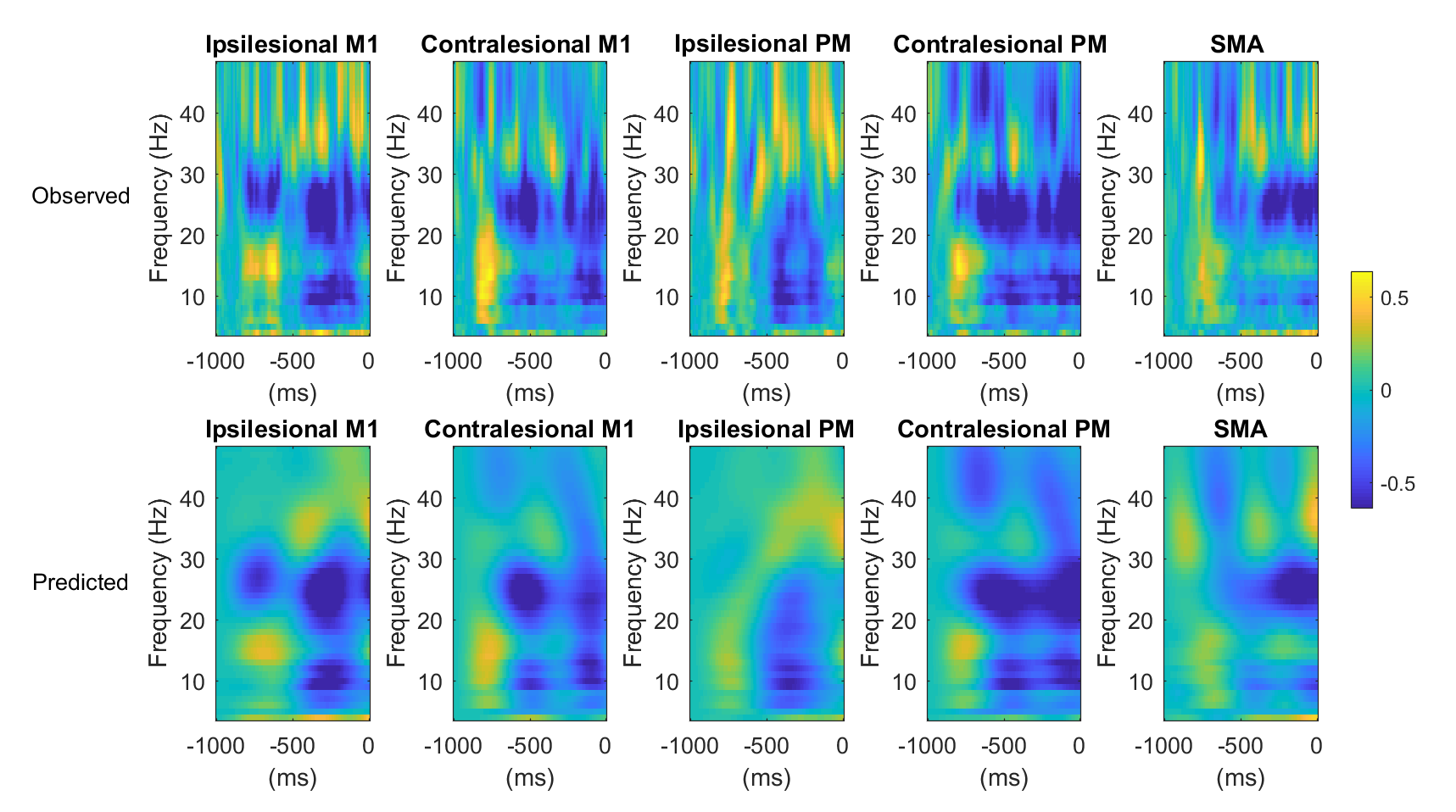


Supplementary Figure 2. The observed (top) and model-predicted (bottom) spectrograms for each region for one participant using the winning model (Model 12). Yellow indicates an increase in power compared to baseline and blue indicates a decrease in power compared to baseline. 0 ms indicates movement onset. Overall, the model explained ~85% of the original spectral variance for each condition.


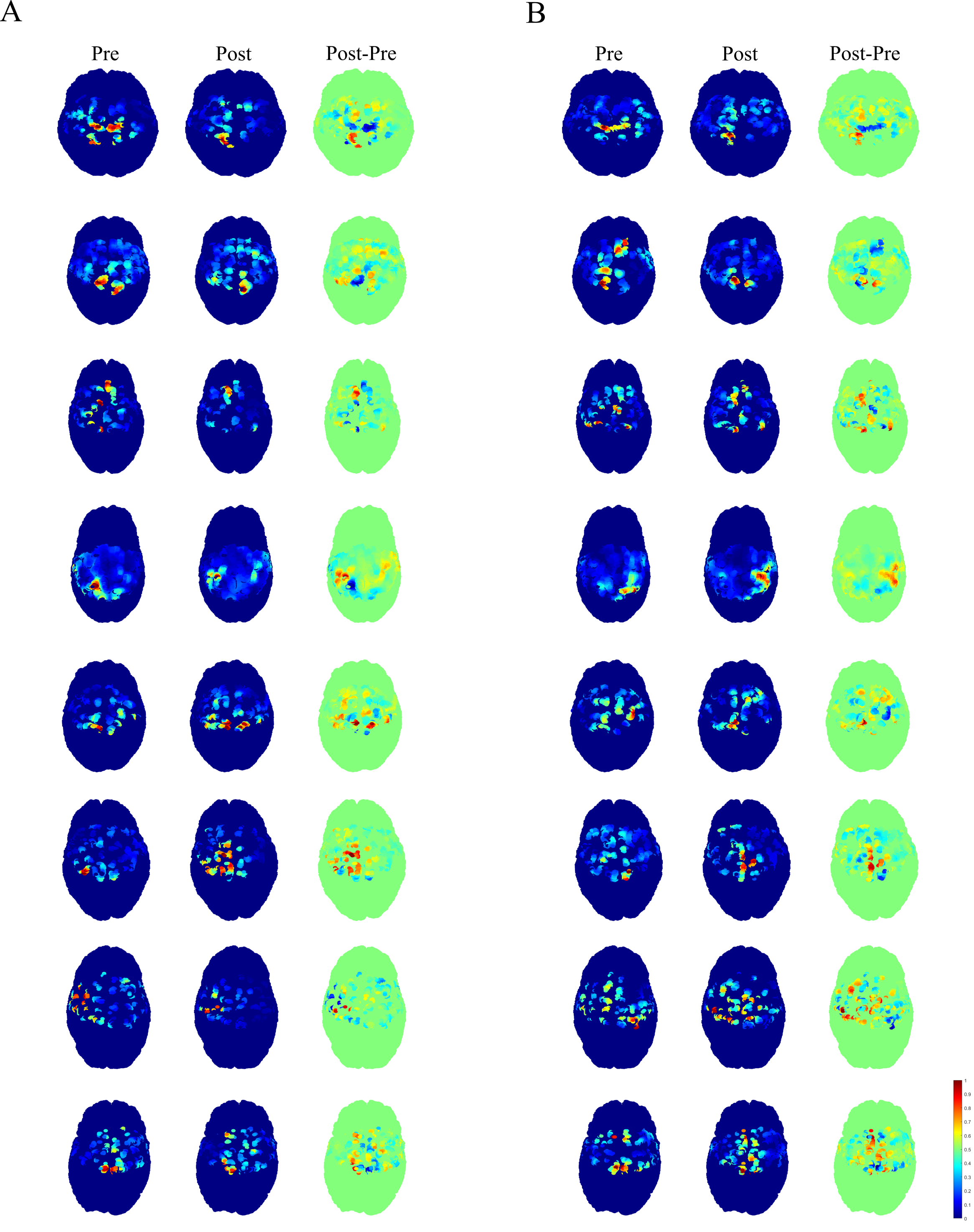


Supplementary Figure 3. Current source density maps for each participant. (A) Current source density maps for Pre-Intervention (Left column), Post-Intervention (Middle column), and the difference between post- and pre-intervention (Right column) for Hand Opening on the table for each participant. (B) Current source density maps for Hand Opening while Lifting for each participant. The color scheme is the same as that used for Figure 5. Red indicates higher activity, while blue indicates lower activity. Lesions were flipped so that the Left hemisphere represents the lesioned hemisphere for each participant.


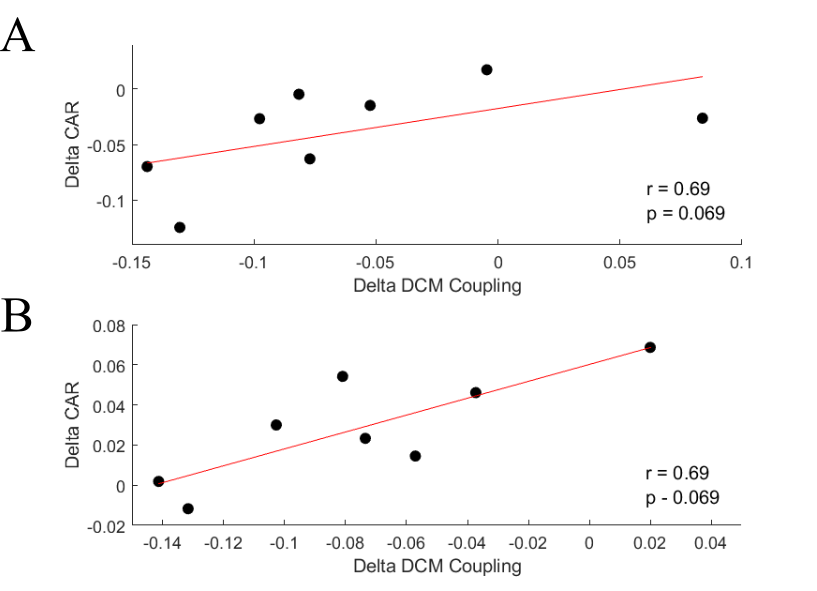


Supplementary Figure 4. Associations between intervention-induced changes in DCM coupling and CAR. (A) Change (Post-Pre) in contralesional M1 to ipsilesional M1 gamma-gamma coupling compared to change in contralesional M1/S1 CAR for Hand Opening. (B) Change in ipsilesional M1 gamma-beta coupling compared to change in ipsilesional M1/S1 CAR for Hand Opening while Lifting.
